# Metabolic Reprogramming of Microglia in Sepsis-Associated Encephalopathy: Insights from Neuroinflammation

**DOI:** 10.2174/1570159X21666221216162606

**Published:** 2023-07-10

**Authors:** Shenjia Gao, Yi Jiang, Zhaoyuan Chen, Xiaoqiang Zhao, Jiahui Gu, Han Wu, Yun Liao, Hao Sun, Jun Wang, Wankun Chen

**Affiliations:** 1 Department of Anesthesiology, Cancer Center, Zhongshan Hospital, Fudan University, 180# Feng-Lin Road, Shanghai, 200032, China;; 2 Shanghai Key Laboratory of Perioperative Stress and Protection, Shanghai, China;; 3 Department of Integrative Medicine and Neurobiology, School of Basic Medical Science, State Key Laboratory of Medical Neurobiology, Fudan University, Shanghai, 200032, China;; 4 Shanghai Medical College of Fudan University, Shanghai, China;; 5 Fudan Zhangjiang Institute, Shanghai, 201203, China

**Keywords:** Sepsis-associated encephalopathy, neuroinflammation, microglia, metabolic reprogramming, sepsis, microglia

## Abstract

Sepsis-associated encephalopathy (SAE) is a diffuse brain dysfunction caused by sepsis that manifests as a range of brain dysfunctions from delirium to coma. It is a relatively common complication of sepsis associated with poor patient prognosis and mortality. The pathogenesis of SAE involves neuroinflammatory responses, neurotransmitter dysfunction, blood-brain barrier (BBB) disruption, abnormal blood flow regulation, *etc*. Neuroinflammation caused by hyperactivation of microglia is considered to be a key factor in disease development, which can cause a series of chain reactions, including BBB disruption and oxidative stress. Metabolic reprogramming has been found to play a central role in microglial activation and executive functions. In this review, we describe the pivotal role of energy metabolism in microglial activation and functional execution and demonstrate that the regulation of microglial metabolic reprogramming might be crucial in the development of clinical therapeutics for neuroinflammatory diseases like SAE.

## 1 INTRODUCTION

Sepsis is a life-threatening organ dysfunction caused by a dysregulated host response to infection [1]. In 2017, there were 48.9 million sepsis cases globally and 11 million sepsis-related fatalities, accounting for 19.7% of the overall global death toll [2]. Sepsis-associated encephalopathy (SAE) is a diffuse brain dysfunction caused by sepsis and an early feature of infection in the body [3]. SAE is found in more than half of septic patients in intensive care units (ICUs), with symptoms ranging from mild delirium to coma [3-5]. It is a major cause of in-hospital mortality and poor prognosis in septic patients [6]. The brain dysfunction in SAE is currently thought to be the consequence of systemic metabolic, inflammatory, and hemodynamic disturbances induced by systemic inflammatory response syndrome (SIRS) rather than direct pathogen infection of the central nervous system (CNS) [7]. Although there is no infection in the brain, peripheral inflammatory signals can generate extensive neuroinflammation *via* neuronal and humoral pathways [8]. There is evidence that the neuroinflammatory process involves endothelial cell activation, blood-brain barrier alterations, cellular dysfunction in microglia and neurons, and alterations in neurotransmission [9, 10].

Microglia, as the main immune cells of the central nervous system, account for 5%-10% of total brain cells and play an important role in neuroinflammation [11, 12]. Studies have shown that in SAE, microglia are in a highly activated state, causing neuronal dysfunction and cognitive memory deficits through the release of large amounts of inflammatory mediators and neuromodulators [11, 13, 14]. Metabolic reprogramming during microglial activation is one of their most prominent features. Metabolic reprogramming refers to the alteration of various intracellular metabolic processes in response to micro-environmental changes [15]. When microglia are stimulated by inflammation, cellular metabolism is reprogrammed from a homeostatic pathway dominated by oxidative phosphorylation (OXPHOS) to aerobic glycolysis, which rapidly meets the metabolic demands of microglia and provides energy for their proliferation, migration, cytokine secretion, and phagocytosis, despite the lower efficiency of glycolysis compared to OXPHOS.

Currently, the LPS injection model and the cecal ligation perforation (CLP) model are commonly used in sepsis research, both of which are effective in inducing short- and long-term behavioral memory deficits in animals [16]. In this review, we mainly assess the activation of microglia in neuroinflammation and their phenotypic transformation, discuss how metabolic reprogramming regulates the activation process and functional performance of microglia, and consider how targeting and regulating microglial metabolism can effectively alleviate neuroinflammation and improve cognition.

## 2 MICROGLIAL ACTIVATION PHENOTYPES AND FUNCTIONAL SUBPOPULATIONS

Microglia, as tissue-resident macrophages in the CNS, express several macrophage-related markers and display a functional polarization similar to that of macrophages. Similar to peripheral macrophages, activated microglia can be classified into M1 and M2 types. LPS and IFN-γ can stimulate the transformation of microglia into the M1 type and exert pro-inflammatory effects [17]. Markers of M1 microglia include MHC-II, CD16, CD32, CD40, CD54, CD80, CD86, and CCR7, which can produce IL-1β, IL-6, TNF-α, CCL2, ROS, NO, glutamate, and other pro-inflammatory cytokines, which induce BBB destruction, leading to neuronal damage and dysfunction [18-21]. IL-4 and IL-13 can convert microglia into M2 types and exert anti-inflammatory and tissue repair effects [17]. M2 microglia express Arg-1, CD36, CD163, CD206, CD200R, and other markers and can synthesize IL-10, transforming growth factor β (TGF-β), brain-derived neurotrophic factor (BDNF), and insulin-like growth factor-1 (IGF-1), acting in an anti-inflammatory fashion and helping to restore homeostasis of the CNS [19-21]. Additionally, M2 microglia can be further divided into M2a, M2b, and M2c subtypes [19]. M2a microglia are mainly involved in cell regeneration and tissue repair, while M2b and M2c cells are mainly involved in phagocytosis and the removal of tissue debris [19].

However, there are still great differences between microglia and macrophages, both in developmental processes and neurobiological functions, and the use of the M1/M2 differentiation model is too simplified and has major limitations in reflecting the response status of microglia [22]. In recent years, the development of cell sequencing technology has inspired further ideas and provided evidence for microglia typing. According to the transcriptional characteristics of microglia in different states, they are divided into proliferative-region-associated microglia (PAM) [23], degenerative disease-associated microglia (DAM) [24], injury-activated microglia (IAM) [25], neurodegenerative microglia phenotype (MGnD) [26], highly activated microglia (HAM) [27], and other functional subpopulations. More studies have been carried out to annotate microglia subsets by single-cell sequencing and other technologies, but no one has further classified microglial functional subpopulations in SAE so far. Therefore, further classification of microglia in SAE is necessary, which is also a very innovative research direction.

Metabolism-related genes are enriched to varying degrees in these microglial subsets. For example, PAM are enriched in all genes, including those responsible for oxidative phosphorylation, glycolysis, and β-oxidation, while genes responsible for lysosomal acidification, lipid transport, and metabolism are also upregulated [23]; DAM express a series of genes related to lipid metabolism, including apolipoprotein E (*ApoE*), lipoprotein lipase (*Lpl*) and *Trem2* [24, 28]. This suggests that metabolic reprogramming plays an important role in the microglial phenotypic transition and functional differentiation [29].

## 3 MICROGLIA ACTIVATION PATHWAYS IN SAE

**Fig. (1):**
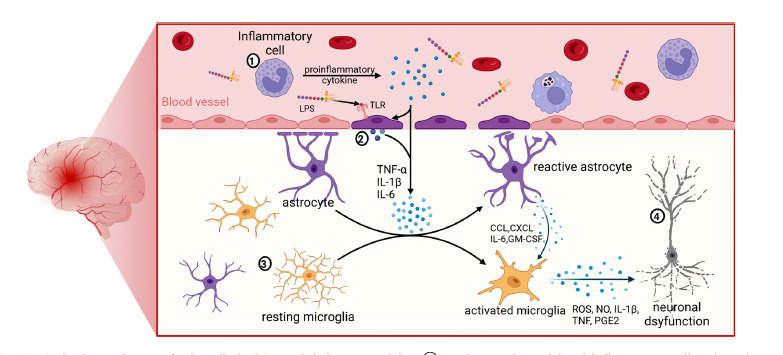
Activation pathways of microglia in SAE and their neurotoxicity. **①** During sepsis, peripheral inflammatory cells release large amounts of inflammatory factors in response to pathogen stimulation. These inflammatory factors and pathogen components (such as LPS) can act on endothelial cells of the BBB through blood circulation, leading to endothelial cell activation. **②** Activated endothelial cells can produce large amounts of inflammatory cytokines acting on the CNS. At the same time, peripheral inflammatory stimulation can also cause endothelial cell apoptosis, resulting in BBB destruction and increased permeability, and peripheral inflammatory factors can thus cross the BBB into the brain, causing astrocyte and microglia activation. **③** Homeostatic microglia are transformed into the M1 phenotype by inflammatory signals and release large amounts of inflammatory mediators. At the same time, activated astrocytes produce CCL, CXCL, IL-6, GM-CSF, and other cytokines, which are involved in regulating microglial migration, activation, and proliferation. **④** ROS, NO, and various cytokines released from M1 microglia are toxic to neighboring neurons, leading to neuronal dysfunction.

Under homeostatic conditions, microglia in the CNS have a branched morphology called homeostatic microglia, and their branches are highly motile and constantly “scan” the surrounding environment and interact with surrounding neurons, astrocytes, and vascular endothelial cells for information exchange to monitor changes in the internal environment and maintain brain homeostasis [30-32]. In response to neuroinflammatory conditions, microglia change from a branching to an “amoeboid” structure with larger cell bodies and shorter branches and are highly activated in phagocytosis and pro-inflammatory functions, exerting neuroprotective or damaging effects (Fig. **1**) [32].

### 3.1 Peripheral Inflammatory Signals

Studies have demonstrated that in SAE, the structure of the BBB is destroyed 4 hours after the onset of peripheral inflammation, and BBB permeability is increased, thus allowing inflammatory cytokines from the peripheral circulation to enter the brain and act on the CNS [33, 34]. Peripheral cytokines, including tumor necrosis factor α (TNF-α), interleukin 1β (IL-1β), and interleukin 6 (IL-6), can induce microglia to differentiate into a proinflammatory state [30]. Previous research by our group has found that 24 hours after abdominal surgical trauma, elevated levels of inflammatory factors in peripheral blood activated microglia by reducing their miR-124 levels [35].

Peripheral inflammatory signals can also be transmitted to the brain *via* the vagus nerve [36]. The vagus nerve is the main afferent nerve pathway from the abdominal cavity to the brain, and cutting off the vagus nerve can eliminate the elevation of IL-1β and TNF-α in the hypothalamus and hippocampus induced by intraperitoneal LPS injection, alleviate LPS-induced inhibition of exploratory and feeding behaviors in mice, and attenuate LPS-enhanced nonrapid eye movement sleep (NREMS) [37, 38].

Significant recruitment of monocytes to the brain has been reported in neuroinflammatory diseases, such as traumatic brain injury, intracerebral hemorrhage, epilepsy, and autoimmune encephalomyelitis [39-42]. However, the specific mechanism by which monocytes impair cognitive function is still unclear, which may involve neuronal demyelination. Recent studies have demonstrated that in sepsis, infiltration of peripheral inflammatory cells into the CNS causes microglia activation, leading to cognitive dysfunction [8]. The infiltration of CCR2^+^ monocytes and macrophages into the CNS plays an important role in SAE-induced long-term cognitive dysfunction, and inhibition of monocyte and macrophage infiltration can further inhibit microglia activation [4, 8].

### 3.2 Central Inflammatory Signals

During the onset of SAE, acute systemic inflammation can rapidly affect the cerebral vasculature. Although structural damage to the BBB takes time to develop once peripheral inflammation begins, cerebrovascular endothelial cells can be triggered early [33]. Endothelial cells can express receptors for IL-1β, IL-6, and TNF-α, and when inflammatory substances from the periphery bind to the receptors on the surface of endothelial cells, they can activate the NF-κB pathway, promote the expression of inflammatory genes, such as *Tnf* and *Tnfaip3*, transmit peripheral inflammatory signals, and further stimulate astrocytes and microglia [33]. Endothelial cells can also recognize circulating LPS *via* toll-like receptors (TLRs), which induce NF-κB pathway activation [43]. Moreover, vascular adhesion molecule 1 (VCAM1) shed by endothelial cells can activate microglia, inhibit neural progenitor cell (NPC) activity, and impair cognition [44].

In addition, astrocytes, one of the components of the BBB, are also involved in microglial activation. Studies have shown that the increased cytokines in the hippocampus between 4 and 24 hours after intraperitoneal LPS injection are mainly produced by astrocytes [36, 45]. Activated astrocytes can produce a large number of chemokines, including chemokine (C-C motif) ligand (CCL) and chemokine (C-X-C motif) ligand (CXCL) [46]. CCL11 can significantly promote microglial migration and activate microglia by upregulating nicotinamide adenine dinucleotide phosphate oxidase (NOX) 1 and producing reactive oxygen species, which cause excitotoxic neuronal death, resulting in behavioral changes and memory impairment [47]. Activated astrocytes can also secrete cytokines, such as IL-6 and GM-CSF, which regulate microglial migration, activation, and proliferation [46].

### 3.3 Neuronal Signals

Neuronal hyperexcitation also activates microglial. Matrix metalloproteinase 9, ATP, and various chemokines (monocyte chemotactic protein 1, CX3CL1, and others) are secreted by activated neurons and regulate microglial migration, activation, and neurotoxicity [48]. Microglia also express a variety of neurotransmitter receptors, including ionotropic glutamate receptors (iGluRs), metabotropic glutamate receptors (mGluRs), adrenergic receptors (ARs), cholinergic receptors, GABA receptors, and dopaminergic receptors, which can recognize neurotransmitters released by neurons and further exert neuroprotective or neurotoxic effects [49]. For example, stimulation of different glutamate receptors in microglia can induce the differentiation of homeostatic microglia into an anti-inflammatory or proinflammatory state, where activation of NMDA receptors and mGLuR I can enhance neurotrophic factor expression in microglia and thus exert neuroprotective effects in neurodegenerative diseases [50], whereas activation of mGLuR II can lead to increased levels of TNF-α expression in microglia and exacerbate neuroinflammation [51]. It was found that activation of adrenergic receptors expressed on microglia after abdominal surgical trauma mediated the neuroinflammatory response [52]. Activated microglia can also express α7 nicotinic acetylcholine receptors (α7nAChR), and the use of acetylcholine and nicotine can inhibit the inflammatory response of microglia induced by LPS, as well as reduce infarct size and functional defects in stroke [53].

## 4 METABOLIC REPROGRAMMING REGULATES THE ACTIVATION OF MICROGLIA

### 4.1 Glycometabolism

#### 4.1.1 Glycolysis

Microglia, like most cells, preferentially employ glucose as a metabolic substrate and generate ATP mainly through OXPHOX [54]. Homeostatic microglia are activated in response to diverse pathogenic stimuli, and their morphology, function, and metabolism are altered accordingly [17].

**Fig. (2):**
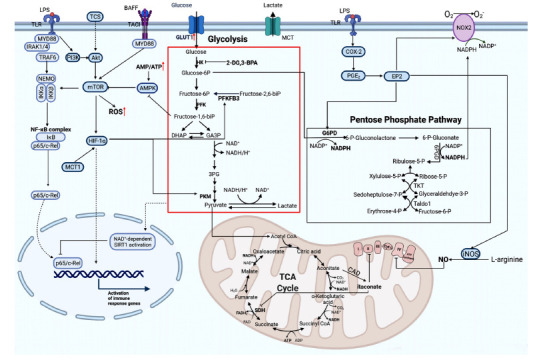
Microglial metabolism shifts from OXPHOX to glycolysis in the inflammatory state. Microglia can recognize PAMPs (*e.g*., LPS) and DAMPs (*e.g*., ATP) as well as various cellular inflammatory factors. Some inflammatory stimuli, including LPS, ATP, INF-γ, and BAFF, can cause enhanced glycolysis in microglia, promoting the transcription of inflammatory genes, which are mainly dependent on the activation of mTOR. Additionally, HIF-1α can promote glycolysis by upregulating the expression of key enzymes of glycolysis. LPS can upregulate the level of mitochondrial itaconate and inhibit SDH/complex II, leading to disruption of the TCA cycle and electron transport chain, which together inhibit mitochondrial OXPHOX. LPS can also upregulate COX-2 and promote the production of PGE2, which can act on EP2 to promote G6PD expression, enhance PPP, and increase NADPH production. EP2 can promote iNOS to produce NO and NOX2, which further generate free radicals. NO can inhibit complex IV of the mitochondrial electron transport chain, and free radicals can act on neighboring neurons and cause neuronal damage. When 2-DG, 3-BPA is used to inhibit HK2 or GLUT1 is silenced to inhibit glycolysis in microglia, the level of fructose 1,6-bisphosphate decreases, the AMP/ATP ratio increases, and the inhibition of AMPK is diminished, leading to AMPK inhibition of the mTOR signaling pathway and suppression of inflammatory gene transcription.

Microglia activated by inflammation switch their energy metabolism from OXPHOX to glycolysis and express high levels of glucose transporter type 1 (GLUT1) to increase the uptake of glucose to meet high levels of glucose demand (Fig. **2**) [55]. When GLUT1 is blocked, microglial glycolysis and phagocytosis are inhibited, and the metabolic pathway can be further reprogrammed to OXPHOX [55]. When microglial glycolysis is inhibited with the glycolysis inhibitors, 2-deoxy-D-glucose (2-DG) and 3-bromopyruvate (3-BPA), or by silencing GLUT1 and hexokinase (HK) 2, the transcriptional activity of NF-κB in microglia is inhibited, thereby blocking microglial activation induced by LPS [56]. This inhibitory effect may involve the AMPK/mTOR/IKKβ and NAD^+^/SIRT1/p65 signaling pathways [56].

The mechanistic target of rapamycin (mTOR) is a serine/threonine kinase that is involved in cellular metabolism. The blockade of the mTOR signaling pathway can shift activated microglia from the pro-inflammatory M1 to the anti-inflammatory M2 subtype [57]. LPS and ATP were found to activate the PI3K/mTOR pathway through TLR and the ATP receptor P2X7 on the surface of microglia, enhancing glycolysis and ROS production and inhibiting the activity of mTOR can inhibit glycolysis and the production of ROS in microglia [58]. In addition to pathogen and injury signals, cytokines can also regulate microglial metabolic reprogramming through mTOR signaling. B-cell activating factor (BAFF) belongs to the tumor necrosis factor (TNF) family, which has an important role in adaptive immunity [59]. Its regulation of microglia also involves mTOR, which can enhance microglial metabolic reprogramming by activating the Akt/mTOR/HIF-1α signaling pathway in microglia, enhancing aerobic glycolysis and increasing lactate production as well as the NAD^+^/NADH ratio [59, 60]. Rapamycin can block mTOR/HIF-1α activation and cellular metabolic reprogramming in microglia [59, 60]. Similar to these results, the broad-spectrum antimicrobial agent triclosan (TCS) also induces a metabolic switch to glycolysis in microglia *via* the Akt/mTOR/HIF-1α signaling pathway, driving its inflammatory activation [61].

Lactate, a special product of glycolysis, also plays an important role in the development of the nervous system. Monocarboxylate transporter (MCT) and lactate dehydrogenase (LDH) are key proteins for lactate transport and oxidation, respectively [62]. MCT1 can enhance the expression of 6-phosphofructo-2-kinase/fructose-2,6-bisphosphatase 3 (PFKFB3) *via* HIF-1α, mediate classical microglial polarization, and further enhance glycolytic flux [63]. Intracerebroventricular administration of exogenous lactate can inhibit LPS-induced microglial activation and reduce neuroinflammation and disease behavior in mice [63]. Additionally, pyruvate kinase (PKM) is a direct HIF-1 target gene, and the positive feedback loop between PKM2 and HIF-1 may accelerate metabolic reprogramming [64].

#### 4.1.2 The Pentose Phosphate Pathway

In response to LPS, microglial metabolism shifts to glycolysis with concomitant upregulation of the pentose phosphate pathway (PPP) and increased fatty acid synthesis (FAS) due to disruption of the tricarboxylic acid cycle (TCA) [65]. PPP is the main source of NADPH and ribose phosphate, and NADPH can be involved in a variety of metabolic reactions as a hydrogen donor, as well as through NOX, to produce ROS; ribose phosphate is involved in the nucleic acid synthesis. The mRNA levels of NOX2 and glucose-6-phosphate dehydrogenase (G6PD) are elevated in microglia after LPS stimulation [66]. When NOX2 is inhibited, microglia can shift from an activated state to an alternative state in response to an inflammatory challenge [67]. G6PD is a key enzyme for the PPP, and abnormal upregulation of G6PD in microglia leads to excessive production of NADPH, which provides an abundant substrate for overactivated NOX2, resulting in high reactive oxygen species (ROS) production and exacerbation of LPS-induced chronic neurodegeneration [68]. Inhibition of G6PD activity ameliorates LPS-induced ROS production and NF-кB activation, thereby inhibiting microglial activation [68].

#### 4.1.3 Mitochondrial Oxidative Phosphorylation

Mitochondria also play an important role in the metabolic reprogramming of microglia. LPS stimulation enhances glucose utilization and lactate production in the brain, and modulation of mitochondrial function in microglia can regulate microglial cell responsiveness to inflammation, such as cytokine release and cell morphology changes [69]. LPS stimulation increases the rate of glycolysis in microglia while preserving oxidative phosphorylation [69]. In contrast, when LPS and IFN-γ act in combination, microglia exhibit enhanced glycolysis and inhibit oxidative phosphorylation [69]. The inducible nitric oxide synthase iNOS is overexpressed in microglia, producing large amounts of nitric oxide (NO) that inhibits complex IV in the electron transport chain, leading to disruption of the electron transport chain and the production of large amounts of oxidants, causing microglia to exhibit potent neurotoxicity [69]. NO produced by microglia can also act on mitochondria in neurons, inhibiting complex IV of the mitochondrial electron transport chain and causing neuronal damage [70]. LPS stimulation also induces the expression of cis-aconitate decarboxylase (CAD), which catalyzes the production of itaconate. Itaconate can inhibit succinate dehydrogenase (SDH/complex II), leading to intracellular succinate accumulation and disruption of the TCA cycle [69]. Itaconate can also exert neuroprotective effects by antagonizing NO metabolism, leading to neurotoxicity in microglia when the itaconate/NO ratio is imbalanced, causing neuronal dysfunction [69]. Exogenous use of itaconate inhibits the inflammatory response of microglia and prevents neurodegeneration [69].

### 4.2 Lipid Metabolism

**Fig. (3):**
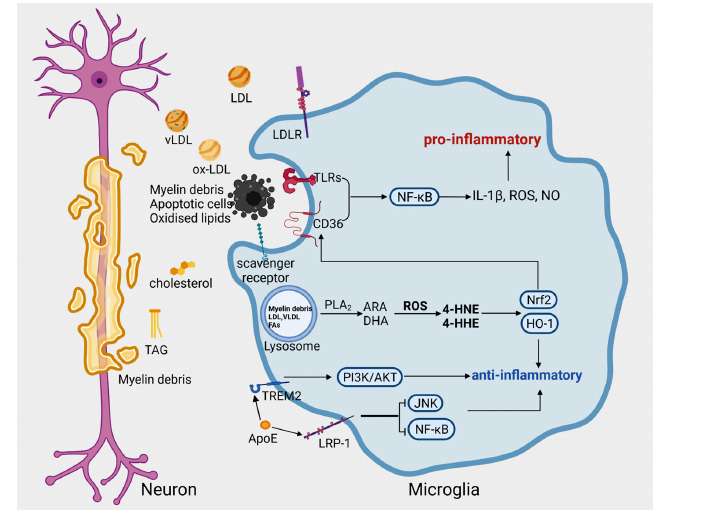
Lipid metabolism in microglia in the inflammatory state. Microglia recognize and phagocytose myelin debris, oxidized lipids, lipoproteins, ApoE, and other substances through surface scavenger receptors (*e.g*., CD36 and LRP1) and lipoprotein receptors (*e.g*., LDLR and VLDLR). CD36 mediates microglial phagocytosis of myelin debris, and its expression level is regulated by NRF2. The synergistic action between CD36 and TLR activates downstream inflammatory signaling of NF-κB and promotes the production of inflammatory mediators (*e.g*., IL-1β, ROS, and NO). ApoE can bind to LRP1 and TREM2 on the surface of microglia to inhibit microglial activation and reduce neuroinflammation. The lipid components phagocytosed by microglia can be hydrolyzed by PLA2 to release ARA and DHA. ARA and DHA can be oxidized by ROS to produce 4-HNE and 4-HHE, respectively, both of which can activate the NRF2 antioxidant pathway and increase HO-1 expression to exert anti-inflammatory and antioxidant effects.

Lipids are the most abundant component of the CNS, accounting for approximately 50% of the dry weight of the brain and comprising structures, including phospholipid bilayers and myelin sheaths. Furthermore, as signaling molecules, lipids play a role in regulating the inflammatory response of microglia. According to research, glucose metabolism is critical in the early phases of microglial activation, while lipid metabolism is more important in the later stages (Fig. **3**) [71].

In multiple sclerosis, which is mainly characterized by neuroinflammation, microglia can recognize and phagocytose extracellular myelin debris through the fatty acid translocase CD36 [72]. In the early stages of the disease, nuclear factor-related factor 2 (NRF2) reduces neuroinflammation by upregulating CD36 expression and inhibiting microglia from shifting to a pro-inflammatory state [72]. Microglia phagocytose large amounts of oxidized lipids and myelin debris in response to neural injury at the beginning of the disease, but as more lipids are phagocytosed, secondary cytotoxic effects are caused, and hyperlipidemia can upregulate CD36 expression and exacerbate ischemic brain injury by promoting CD36-mediated inflammation [73]. While CD36 mediates phagocytosis of oxidized phospholipids and apoptotic cells, it can interact with Toll-like receptor family members to form a complex that jointly initiates downstream signaling [74]. CD36 recognition of oxidized low-density lipoprotein (ox-LDL) and β-amyloid peptides can promote the formation of CD36-TLR4-TLR6 heterotrimers that mediate the release of inflammatory mediators (*e.g*., IL-1β, ROS, and NO) associated with ox-LDL and β-amyloid peptides [74].

After microglia engulf myelin debris, they are metabolized *via* enzyme-dependent or non-enzymatic pathways [75]. Myelin sheaths are rich in polyunsaturated fatty acids (PUFAs) [76], such as arachidonic acid (ARA) and docosahexaenoic acid (DHA), which can be released by phospholipase A2 (PLA2) hydrolysis and thus exert metabolic activity [77]. They can also be oxidized by intracellular ROS to generate lipid peroxides [77]. For example, ARA oxidation produces 4-hydroxynonenal (4-HNE), and DHA produces 4-hydroxy-2-hexenal (4-HHE) [77]. Omega-3 PUFAs regulate the oxidative-antioxidative balance in the brain by regulating the NRF2 antioxidant pathway and heme oxygenase-1 (HO-1) expression. DHA and its lipid peroxidation product 4-HHE have been shown to attenuate LPS-induced upregulation of NO, ROS, and p-cPLA2 expression, exerting antioxidant and anti-inflammatory effects [77, 78]. Other unsaturated fatty acids (UFAs) in the brain, such as linoleic acid (LA), oleic acid (OA), and α-linolenic acid (ALA), were shown to protect microglia and ameliorate the reduced cell viability caused by the lipotoxicity of palmitic acid [79, 80].

ARA is converted to prostaglandins (PG) and other lipid mediators by cyclooxygenase (COX) [81]. Prostaglandin E2 (PGE2) attenuates LPS-induced TNF-α expression by acting on E-type prostanoid receptor 4 (EP4) in naïve microglia [66]. However, in classically activated microglia, EP4 expression is reduced, and EP2 expression is increased [66]. EP2 regulates the expression of metabolism-related genes, such as iNOS and NOX2, and promotes metabolic conversion in microglia [66]. In aging microglia, PEG2 promotes the synthesis of glycogen *via* EP2, reducing glucose flux and mitochondrial respiration and causing an abnormal inflammatory response [82].

Apolipoprotein E (ApoE) is a glycosylated protein whose main function is to transport lipids in the circulatory system and between cells, and microglia can recognize and phagocytose ApoE through the low-density lipoprotein receptor (LDLR), very low-density lipoprotein receptor (VLDLR), and LDL receptor-related protein-1 (LRP1) [83]. ApoE has multiple neuroprotective effects, including anti-inflammatory, anti-apoptotic, and antioxidant effects, as well as maintenance of cerebrovascular integrity [84]. Studies have demonstrated that LRP1, one of the ApoE receptors, inhibits the activation of microglia by regulating the JNK and NF-κB signaling pathways [85, 86] and promotes microglia to acquire an anti-inflammatory phenotype through the Shc1/PI3K/Akt signaling pathway [84]. Additionally, ApoE is a novel, high-affinity ligand for TREM2 discovered in recent years. An ApoE-mimetic peptide can activate TREM2 to inhibit the activation of microglia after intracerebral hemorrhage and attenuate neuroinflammation and neuronal apoptosis through activation of the PI3K/Akt signaling pathway [87]. In addition, overexpression of LDLR and knockdown of the *Apoe* gene can promote the development of microglia in the direction of catabolism, upregulate genes encoding ion channels and neurotransmitter receptors, and inhibit microglial activation [88].

Lipoprotein lipase (LPL) plays an important role in lipid metabolism. It is involved in the hydrolysis of core triglycerides (TGs) in chylomicrons and very low-density lipoproteins (VLDLs) and can also interact with lipoproteins to promote their uptake [89]. In the CNS, LPL is highly expressed in microglia, and LPS treatment downregulates the expression of the *Lpl* gene in these cells [90]. Knockdown of the *Lpl* gene significantly reduces the expression of genes associated with the anti-inflammatory function of microglia and causes a shift in lipid availability and substrate metabolism, suggesting that LPL is essential for microglial phenotypic transition [90]. LPL expression is also influenced by age, and a type of highly activated microglia (HAM) was found in the brains of aged mice, which characteristically overexpressed *Lpl* and *Lgals3* (encoding galactose lectin-3), thus mediating cell survival, energy metabolism, and immune-inflammatory responses [27]. Moreover, LPL-deficient microglia were found to exhibit impaired immunoreactivity and phagocytosis, mitochondrial metabolic substrate conversion to glutamine, and mitochondrial dysfunction [91].

### 4.3 Amino Acid Metabolism

Amino acids are the basic building blocks of proteins, and one of their important physiological functions is to participate in protein synthesis. However, when sugar metabolism or lipid metabolism cannot meet the energy requirements of cells, they can also use proteins as their energy source. Microglia can use glutamine for energy metabolism when there is a lack of glucose in the environment, and mTOR-mediated signaling pathways play an important role in this process [92]. Glutamine or glutamate catabolism can activate mTOR and enhance the response of mTORC1 to amino acids. During this process, mTOR can sense the availability of cellular energy and provide feedback through glutamine [93, 94].

Homocysteine (Hcy) is a sulfur-containing amino acid produced during methionine metabolism. Hcy participates in the methionine cycle and is involved in numerous methylation reactions in the body [95, 96]. Hcy can also exert various physiological effects after being converted to cysteine through transsulfuration [95, 96]. Low doses of Hcy can exert pro-inflammatory effects by upregulating microglial NADPH oxidase activity, enhancing ROS production, and promoting glial cell proliferation and activation [97]. The JAK/STAT pathway and the p38 MAPK pathway play important roles in Hcy-mediated microglial activation [91, 92]. Reducing Hcy levels in the brain can reduce the activation of microglia and the release of pro-inflammatory factors [98].

Microglia also express all the enzymes in the kynurenine pathway (KP), a major degradation pathway for tryptophan (Trp) and one of the major regulatory pathways of the immune response [99]. The intermediates of KP, quinolinic acid (QUIN) and 3-hydroxykynurenine (3-HK), are neurotoxic and can cause neuronal apoptosis and neurodegeneration, which can be caused by activated microglia and infiltrating macrophages [100]. Under physiological conditions, KP produces kynurenic acid (KYNA), picolinic acid, and NAD+, but under inflammatory conditions, KP overexpresses QUIN and other neurotoxic or inflammatory molecules [99]. Inflammatory mediators, such as IFN-γ, TNF-α, and LPS, can also initiate KP by activating indoleamine 2,3-dioxygenase 1 (IDO-1) in microglia [99].

The studies mentioned above suggest that amino acid metabolism during inflammation has important effects on both microglial activation and function. However, thus far, there are still few studies on this topic, and the specific principles and mechanisms require further study.

## 5 TARGETING MICROGLIAL METABOLISM FOR THE TREATMENT OF SAE

Metabolic reprogramming can govern microglial activity, and several medications are already available to modify the metabolic reprogramming of microglia (Table **1**) [101-107].

2-DG is a glucose analog, whereas 3-BP is a simple lactate analog (a brominated derivative of pyruvate), both of which limit glycolysis by inhibiting hexokinase, and they are mainly used in cancer therapy [108]. At the same time, several investigations have indicated that inhibiting microglial glycolysis with 2-DG or 3-BP can reduce the production of pro-inflammatory molecules, suppress neuroinflammation, and lessen neuronal damage [56, 101].

Pioglitazone and rosiglitazone are peroxisome proliferator-activated receptor gamma (PPARγ) agonists that can modulate mitochondrial complex I activity by increasing the expression of PGC-1α (peroxisome proliferator-activated receptor-γ coactivator-1α) and UCP2 (uncoupling protein-2), reducing LPS-induced lipid peroxidation, and inhibiting microglial activation, thereby exerting antioxidant and anti-inflammatory effects [102]. Rosiglitazone was also found to reverse lipid droplet accumulation in *Lpl*-deficient microglia [28]. In other diseases, such as depression and dementia, PPARγ agonists can differentially modulate microglial activation and neuroinflammation [109, 110].

Aldose reductase (AR), a rate-limiting enzyme of the polyol pathway that reduces glucose to sorbitol, is a therapeutic target in a variety of inflammatory diseases, such as sepsis and ulcerative colitis [111]. The Aldose reductase inhibitors (ARIs), namely Sorbinil (Sor) and Zopolrestat (Zol), can inhibit neuroinflammation by modulating ROS/ PKC-dependent NF-κB and MAPK signaling pathways and significantly inhibiting the production of TNF-α, IL-1β, and IL-6 in microglia [103]. In addition, inhibition of AR leads to the accumulation of 4-HNE in microglia, which induces phosphorylation of cAMP response element binding protein (CREB), inhibits the conversion of microglia to the M1 subtype, promotes the expression of M2-related genes, and exerts anti-inflammatory effects [112].

Rutin is a multifunctional natural flavonoid glycoside that exhibits antioxidant and anti-inflammatory activities in diabetes, obesity, and AD [113]. Sodium rutin (NaR) is its sodium salt form, which has improved water solubility and bioavailability [104]. It has been shown that NaR treatment rescues the metabolic reprogramming of microglia caused by inflammation, enhances OXPHOS in microglial mitochondria, increases microglial phagocytosis receptor expression, and reduces neuroinflammation [104].

Epidemiological evidence suggests that diets rich in PUFAs have anti-inflammatory effects, and DHA and eicosapentaenoic acid (EPA) are the main active components of ω-3 PUFA [105]. DHA can inhibit oxidative stress and pro-inflammatory responses in microglia by promoting the expression of HO-1 [105]. In addition, metabolites of ω-3PUFA, including epoxyeicosatetraenoic acid-ethanolamide (EEQ-EA), epoxydocosapentaenoic acid-ethanolamide (EDP-EA), 4-HHE, and 4-HNE, can exert anti-inflammatory effects and can be used as potential therapeutic targets for neuroinflammation [77, 78, 105, 114].

Choline is a precursor of the neurotransmitters, acetylcholine and phosphatidylcholine, and it can convert homocysteine to methionine by providing a methyl group through betaine, a metabolite of choline [98]. Choline supplementation can alter the expression of genes related to the immune response, histone modification, and neuronal death regulation in the brain and reduce the activation of microglia in neuroinflammation [98].

Fisetin, an effective ingredient of Cotinus coggygria, is used in the treatment of many neurological disorders. Recent studies have demonstrated that Fisetin has a therapeutic effect on cognitive dysfunction caused by SAE. It can reduce SAE-induced cognitive dysfunction by inducing mitochondrial autophagy and scavenging reactive oxygen species [106].

(-)-Epicatechin is a natural polyphenolic substance from dietary flavonoids. It has been shown to modify the metabolic profile and blood's rheological properties and cross the blood-brain barrier [115]. In SAE, administration of (-) -epicatechin can reduce cognitive decline and neuronal damage and prevent neuronal dendritic spine loss. This suggests that (-)-epicatechin treatment may be a promising treatment for improving cognitive function in patients with sepsis [107].

## CONCLUSION

SAE is a common complication of sepsis and an important factor in the prognosis of septic patients. As sentinels and guards of the central nervous system, microglia play a vital role in SAE. The energy consumption of microglia varies with cell phenotype, and metabolic reprogramming, as a fundamental driver of the microglial immune response, plays a decisive role in the phenotypic conversion and function of microglia. Immunometabolism of microglia is increasingly recognized as a key hub in controlling the immunological state of the CNS. However, most of the data gathered in the current investigations are from microglia cultivated *in vitro*, and the existing information implies that the metabolic status of microglia *in vivo* is temporal, spatial, and even sex-dependent. The role of different metabolic states of microglia in SAE and other CNS inflammatory diseases warrants further investigation. Altering the immunophenotype of microglia during disease by targeting metabolic reprogramming may be an effective therapeutic strategy.

## Figures and Tables

**Table 1:** The drugs for regulating metabolic reprogramming of SAE.

**Drugs**	**Disease**	**Target**	**Effects**	**ClinicalTrials.gov Identifier**	**Diseases Corresponding to Clinical Trials**	**References**
2-DG	AD	Hexokinase	Inhibits glycolysis	NCT00096707	Cancer	[101]
3-BPA	PD	Hexokinase	Inhibits glycolysis	-	-	[56]
Pioglitazone	SAE	PPARγ (agonist)	Protects mitochondrial function	NCT00982202	AD	[102]
Rosiglitazone	AD	PPARγ (agonist)	Reduces lipid droplet accumulation in microglia	NCT00265148	AD	[28]
Sor and Zol	AD	AR (inhibitor)	Reduces intracellular ROS production	NCT00000159	Diabetic retinopathy	[103]
NaR	AD	-	Enhances OXPHOS	-	-	[104]
DHA	Depression	HO-1	Inhibits oxidative and pro-inflammatory responses in microglia	NCT00772096	SAE	[105]
Choline	AD	-	Reduces brain Hcy levels	NCT05021211	AD	[98]
Fisetin	SAE	Pink1/Parkin	Activates mitophagy and suppresses neuroinflammation	-	-	[106]
(-)-Epicatechin	SAE	AMPK	Reduces neuroinflammation, protects mitochondria function	NCT03035201	Cognitive impairment	[107]

**Abbreviations:** 2-DG, 2-Deoxy-D-glucose; 3-BPA, 3-Bromopyruvate; Sor, Sorbinil; Zol, Zopolrestat; NaR, Sodium Rutin; DHA, Docosahexaenoic acid; AD, Alzheimer's Disease; PD, Parkinson's disease ; SAE, Sepsis-Associated Encephalopathy; PPARγ, Peroxisome Proliferator-activated Receptor-gamma; AR, Aldose Reductase; HO-1, Heme Oxygenase 1; ROS, Reactive Oxygen Species; OXPHOS, Oxidative Phosphorylation; Hcy, Homocysteine.

## LIST OF ABBREVIATIONS

AD: Alzheimer’s Disease
ALA: α-Linolenic Acid
ApoE: Apolipoprotein E
AR: Aldose Reductase
ARA: Arachidonic Acid
Arg-1: Arginase-1
ARIs: Aldose Reductase Inhibitors
ARs: Adrenergic Receptors
BAFF: B-Cell Activating Factor
BBB: Blood-Brain Barrier
BDNF: Brain-Derived Neurotrophic Factor
3-BPA: 3-Bromopyruvate
CAD: Cis-aconitate Decarboxylase
CCL: Chemokine (C-C Motif) Ligand
CLP: Cecal Ligation Perforation
CNS: Central Nervous System
COX: Cyclooxygenase
CREB: cAMP Response Element Binding Protein
CXCL: Chemokine (C-X-C Motif) Ligand
DAM: Degenerative Disease-Associated Microglia
2-DG: 2-Deoxy-D-glucose
DHA: Docosahexaenoic Acid
EDP-EA: Epoxydocosapentaenoic Acid-Ethanolamide
EEQ-EA: Epoxyeicosatetraenoic Acid-Ethanolamide
EP: E-Type Prostanoid Receptor
EPA: Eicosapentaenoic Acid
FAS: Fatty Acid Synthesis
G6PD: Glucose-6-Phosphate dehydrogenase
GABA: Gamma-aminobutyric Acid
GLUT1: Glucose Transporter Type 1
HAM: Highly Activated Microglia
Hcy: Homocysteine
4-HHE: 4-Hydroxy-2-hexenal
3-HK: 3-Hydroxykynurenine
HK: Hexokinase
4-HNE: 4-Hydroxynonenal
HO-1: Heme Oxygenase-1
IAM: Injury-Activated Microglia
ICUs: Intensive Care Units
IDO-1: Indoleamine 2,3-Dioxygenase 1
IFN-γ: Interferon-γ
IGF-1: Insulin-Like Growth Factor-1
iGluRs: Ionotropic Glutamate Receptors
IL: Interleukin
iNOS: Inducible Nitric Oxide Synthase
KP: Kynurenine Pathway
KYNA: Kynurenic Acid
LA: Linoleic Acid
LDH: Lactate Dehydrogenase
LDLR: Low-Density Lipoprotein Receptor
LPL: Lipoprotein Lipase
LPS: Lipopolysaccharide
LRP1: LDL Receptor-Related Protein-1
MCT: Monocarboxylate Transporter
mGluRs: Metabotropic Glutamate Receptors
MGnD: Neurodegenerative Microglia Phenotype
mTOR: Mechanistic Target of Rapamycin
NaR: Sodium Rutin
NMDA: N-methyl-D-aspartate
NO: Nitric Oxide
NOX: Nicotinamide Adenine Dinucleotide Phosphate Oxidase
NPC: Neural Progenitor Cell
NREMS: Non-Rapid Eye Movement Sleep
OA: Oleic Acid
ox-LDL: Oxidized Low-Density Lipoprotein
OXPHOS: Oxidative Phosphorylation
PAM: Proliferative-Region-Associated Microglia
PFKFB3: 6-Phosphofructo-2-Kinase/Fructose-2,6-Bisphosphatase 3
PG: Prostaglandin
PKM: Pyruvate Kinase
PLA2: Phospholipase A2
PPARγ: Peroxisome Proliferator-Activated Receptor Gamma
PPP: Pentose Phosphate Pathway
PUFAs: Polyunsaturated Fatty Acids
QUIN: Quinolinic Acid
ROS: Reactive Oxygen Species
SAE: Sepsis-Associated Encephalopathy
SDH: Succinate Dehydrogenase
SIRS: Systemic Inflammatory Response Syndrome
Sor: Sorbinil
TCA: Tricarboxylic Acid Cycle
TCS: Triclosan
TGF-β: Transforming Growth Factor β
TGs: Triglycerides
TLRs: Toll-Like Receptors
TNF: Tumor Necrosis Factor
Trp: Tryptophan
UFAs: Unsaturated Fatty Acids
VCAM1: Vascular Adhesion Molecule 1
VLDLR: Very Low-Density Lipoprotein Receptor
Zol: Zopolrestat
α7nAChR: α7 Nicotinic Acetylcholine Receptors
